# Reinventing the nucleic acid vaccine with self-amplifying RNA

**DOI:** 10.1186/1742-4690-9-S2-O68

**Published:** 2012-09-13

**Authors:** AJ Geall, GR Otten, A Hekele, W Bogers, H Oostermeijer, P Mooij, K Gerrit, E Verschoor, K Banerjee, Y Cu, CW Beard, LA Brito, JB Ulmer, CW Mandl, SW Barnett

**Affiliations:** 1 Novartis Vaccines and Diagnostics, Cambridge, MA, USA; 2 BPRC, Rijswijk, the Netherlands

## Background

Self-amplifying RNAs (replicons) of positive-strand viruses such as alphaviruses are potentially safe and useful vectors for delivering vaccine antigens. We previously showed that recombinant alphavirus replicon particles (VRP), used in prime-boost regimen with Env in MF59 protein protected rhesus macaques against mucosal challenge with SHIVSF162P4 (J. Virol. 84:5975, 2010).

Novartis recently developed a synthetic self-amplifying mRNA (SAM™) vaccine platform, using cell-free RNA production and non-viral vaccine delivery systems. The SAM™ platform avoids the limitations of cell culture production that complicate production of the alphavirus VRPs and other viral vector systems. Safety concerns associated with the potential generation of replication competent virus (RCV) are eliminated, and the absence of viral structural proteins reduces issues associated with anti-vector immunity, a major limitation of other vectored vaccine systems.

## Methods

HIV- SAM™ vaccines were evaluated in small animals and nonhuman primates (NHP).

## Results

Here we show that SAM™ vaccines encoding HIV antigens induced potent systemic and mucosal immune responses in small animals and nonhuman primates (NHP). Humoral and cellular responses elicited in mice with HIV-SAM™ vaccines were superior to naked DNA or RNA and comparable to those seen with alphavirus replicon particles (VRPs). Robust binding and neutralizing antibodies were seen following two immunizations with a HIV-SAM™ vaccine encoding subtype C TV1 gp140 in rabbits. The same vaccine elicited both IFNγ and IL2 T-cell responses, B-cell ELISpots, and Env-specific antibody responses in rhesus macaques, also after only two immunizations. Importantly, the vaccines were well-tolerated with no local or systemic adverse events observed.

## Conclusion

These results provide the first evidence in a primate species that vaccination with formulated self-amplifying RNA is safe and immunogenic, eliciting robust immune responses.

The safety, immunogenicity, and ease of production provided by SAM™ vaccines provide a rationale for accelerated evaluations of this platform in the context of HIV vaccines.

